# The Identification and Conservation of Tunicaminyluracil-Related Biosynthetic Gene Clusters in Several *Rathayibacter* Species Collected From Australia, Africa, Eurasia, and North America

**DOI:** 10.3389/fmicb.2019.02914

**Published:** 2020-01-10

**Authors:** Matthew A. Tancos, Aaron J. Sechler, Edward W. Davis, Jeff H. Chang, Brenda K. Schroeder, Timothy D. Murray, Elizabeth E. Rogers

**Affiliations:** ^1^Foreign Disease-Weed Science Research Unit, United States Department of Agriculture-Agricultural Research Service, Frederick, MD, United States; ^2^Department of Botany and Plant Pathology, Oregon State University, Corvallis, OR, United States; ^3^Department of Entomology, Plant Pathology and Nematology, University of Idaho, Moscow, ID, United States; ^4^Department of Plant Pathology, Washington State University, Pullman, WA, United States

**Keywords:** *Rathayibacter*, toxicus, iranicus, agropyri, tunicaminyluracil, tunicamycin, *Anguina* nematode

## Abstract

Tunicaminyluracil antibiotics are a novel class of toxigenic glycolipids that are synthesized by several soil-associated *Actinomycetes*. The acquisition of a tunicaminyluracil biosynthetic gene cluster (TGC) in *Rathayibacter toxicus* has led to the emergence of the only described, naturally occurring tunicaminyluracil-associated mammalian disease, annual ryegrass toxicity of livestock. Endemic to Australia, *R. toxicus* is obligately vectored by Anguinid seed gall nematodes to the developing seedheads of forage grasses, in which the bacteria synthesize tunicaminyluracils that may subsequently be consumed by livestock and result in high rates of mortality and morbidity. The potential impact of *R. toxicus* on U.S. agriculture has led the U.S. Department of Agriculture – Animal and Plant Health Inspection Service to list *R. toxicus* as a Plant Pathogen Select Agent. *R. toxicus* is the only characterized phytopathogenic bacterium to produce tunicaminyluracils, but numerous *R. toxicus*-like livestock poisonings outside Australia suggest additional bacterial sources of tunicaminyluracils may exist. To investigate the conservation of the TGC in *R. toxicus* and whether the TGC is present in other *Rathayibacter* species, we analyzed genome sequences of members of the *Rathayibacter* genus. Putative TGCs were identified in genome sequences of *R. toxicus, R. iranicus*, *R. agropyri*, and an undescribed South African *Rathayibacter* species. In the latter three species, the putative TGCs have homologs of tunicaminyluracil-related genes essential for toxin production, but the TGCs differ in gene number and order. The TGCs appear at least partially functional because in contrast to atoxigenic species, TGC-containing *Rathayibacter* species were each able to tolerate exogenous applications of tunicamycin from *Streptomyces chartreusis*. The North American *R. agropyri* TGC shows extensive diversity among the sequenced isolates, with presense/absense polymorphisms in multiple genes or even the whole TGC. *R. agropyri* TGC structure does not appear to correlate with date or location of isolate collection. The conservation and identification of tunicaminyluracil-related gene clusters in three additional *Rathayibacter* species isolated from South Africa, the Middle East, and the United States, suggests a wider global distribution of potentially neurotoxigenic plant-associated bacteria. This potential for additional endemic and exotic toxigenic *Rathayibacter* species could have widespread and severe implications for agriculture.

## Introduction

*Rathayibacter toxicus* is a nematode-transmitted Actinobacterium that is endemic to Australia and infects a variety of forage grasses through its close association with Anguinid seed gall nematodes (Price et al., 1979; Bird, 1981). Due to its ability to synthesize a neurotoxigenic tunicaminyluracil antibiotic, it was listed as a Plant Pathogen Select Agent by the U.S. Department of Agriculture – Animal and Plant Health Inspection Service in 2008 (Murray et al., 2015). Animals that consume *R. toxicus*- infected plant material develop bacterial toxicosis-related symptoms, which are referred to as “annual ryegrass toxicity” or “flood plain staggers” (McKay et al., 1993). Poisoned animals display a variety of neurological symptoms including convulsions, paralysis, excessive salivation, muscle and head tremors, abortions, and death (Bourke et al., 1992; Finnie, 2006).

Mammalian disease symptoms are mainly neurological due to the toxin inhibiting *N*-linked glycosylation, which impairs the cardiovascular system and leads to oxygen deprivation and tissue damage (Jago et al., 1983; Finnie and Jago, 1985; Bourke et al., 1992; Finnie, 2006). Bacterial toxicosis caused by *R. toxicus* poisoning has resulted in severe losses for the Australian livestock industry. From 1970 to 1974, 58 outbreaks were reported in Western Australia, which resulted in the loss of thousands of sheep and cattle with morbidity and mortality rates reaching 100 and 77%, respectively (Berry and Wise, 1975). Similar devastating outbreaks reoccurred in the early 1990s in New South Wales, Australia, with the deaths of thousands of grazing livestock (Davis et al., 1995). The association of toxicosis to *R. toxicus* has only been implicated in Australia, but other undiagnosed *R. toxicus*-like poisoning events have been reported outside the country (Mullins, 1941; Haag, 1943; Shaw and Muth, 1949; Cunningham and Hartley, 1959; Galloway, 1961; Schneider, 1981).

In South Africa, between 1979 and 1980, *R. toxicus*-like poisoning symptoms were observed in sheep and cattle that were fed plant material infected with nematode and bacterial galls, from which a *Rathayibacter* species was isolated (Schneider, 1981). In 2009, several horses died in the same Western Cape Province of South Africa after consuming infected plant material (Grewar et al., 2009). A suspected toxigenic *Rathayibacter* species was isolated from dune grass (*Ehrharta villosa* var. *villosa*) leaf galls in 2003 from the same South African region and labeled as ‘*woodii*’ due to its co-isolation and association with the leaf gall nematode *Anguina woodi* (Riley and Swart, 2004; Riley et al., 2004; Murray et al., 2017). Additional livestock poisonings, with symptoms similar to *R. toxicus* poisoning, were documented in New Zealand and the United States during the mid-twentieth century (Mullins, 1941; Haag, 1943; Shaw and Muth, 1949; Cunningham and Hartley, 1959; Galloway, 1961). Several livestock neurological poisonings in Oregon between 1943–1961 were associated with grasses contaminated with *Anguina* species galls, but no toxigenic bacteria were identified or confirmed to be the causative agents in these cases (Haag, 1943; Shaw and Muth, 1949; Galloway, 1961; Jensen, 1961).

Tunicaminyluracils are a novel class of toxigenic nucleoside antibiotics that possess a unique 11-carbon tunicamine backbone, *N*-acetylglucosamine, uracil, and an assortment of fatty acid chains that vary amongst members in the class (Takatsuki et al., 1971; Edgar et al., 1982; Chen et al., 2016). Tunicaminyluracil antibiotics possess broad biological activity against prokaryotes and eukaryotes and inhibit bacterial cell wall biosynthesis and protein glycosylation, respectively (Takatsuki et al., 1971; Tkacz and Lampen, 1975). The ability to synthesize these unique and toxic antibiotics appears to be limited to soil-associated *Actinomycetes*, notably saprophytic *Streptomyces* species. Recently, a putative tunicaminyluracil biosynthetic gene cluster (TGC) was identified in the genome sequences of *R. toxicus* (Sechler et al., 2017). ‘Tunicamycin’ refers to the specific toxin produced by *Streptomyces chartreusis*; therefore the terms ‘tunicaminyluracil’ and ‘tunicaminyluracil antibiotic’ are used here to describe toxins produced by *Rathayibacter* since their exact structure is not known.

*Rathayibacter toxicus* persists in both soil and plant environments, the latter of which exposes livestock to an otherwise soil-associated antibiotic. *R. toxicus* is obligately vectored and requires a successful nematode infestation before bacterial colonization of the plant and developing seed head can occur. Following seasonal rains, dormant nematodes and bacteria rehydrate and emerge from their overwintering galls. Juvenile nematodes migrate toward emerging grass seedlings and *R. toxicus* can adhere to the nematode cuticle and co-colonize the developing grass ovules (Price et al., 1979). As the nematodes complete their lifecycles by modifying nascent ovules into nematode galls, *R. toxicus* may outcompete the nematodes and transform the modified seed gall into a toxigenic bacterial gall (Price et al., 1979; Stynes and Bird, 1982). Both the nematode and bacterial galls are capable of persisting in extreme environments for decades until favorable environmental conditions arise (Murray et al., 2015). The complex, nematode-dependent lifecycle presents many challenges to studying *R. toxicus.* Moreover, molecular investigations are exceedingly difficult to conduct because *R. toxicus* is listed as a biological select agent, recalcitrant to genetic modification, and averse to tunicaminyluracil production *in vitro* (Payne and Cockrum, 1988).

Here, we investigated (i) if TGCs are unique to *R. toxicus* or present in other grass-associated *Rathayibacter* species and determined (ii) the prevalence and diversity of the TGC within *Rathayibacter* species. Genomic analyses of globally collected *Rathayibacter* species identified three previously unknown tunicaminyluracil-related gene clusters in *R. iranicus*, the undescribed South African *Rathayibacter* species ‘*woodii*,’ and the North American species, *R. agropyri*. The TGCs have high sequence similarity to the TGC-essential genes of *R. toxicus* and *Streptomyces chartreusis*.

## Materials and Methods

### Bacterial Strains, Growth Conditions, and DNA Extraction

*Rathayibacter* species evaluated are listed in Table 1. Cultures were preserved in 15% glycerol and stored at −80°C for long term storage. Depending upon the species, *Rathayibacter* strains were incubated for 3–6 days at 25-28°C on modified YGM media (Sechler et al., 2017). The MasterPure Gram-Positive DNA Purification Kit (Epicentre, Madison, WI, United States) was used to extract genomic DNA according to the manufacturer’s protocol, and the DNA was quantified with a Nanodrop-2000 (Thermo Fisher Scientific, Waltham, MA, United States).

**TABLE 1:** Overview of the putative tunicaminyluracil gene clusters (TGC) evaluated in the current study.

**Species**	**No. of sequences analyzed**	**No. of TGC genes^a^**	**TGC size (bp)^b^**	**GC content of TGC (%)**	**GC content of genome (%)**	**MIC (μg/mL)^c^**	**References**
*Rathayibacter toxicus*	26	14	13,402	52	61.5	8.0	Arif et al., 2016; Sechler et al., 2017; Davis et al., 2018
*Rathayibacter iranicus*	6	16	14,104	56	67.1	8.0	This study and Davis et al., 2018
*Rathayibacter agropyri*	12	14	14,067	56	68.0	Variable^d^	This study and Davis et al., 2018
*Rathayibacter woodii*	1	14	16,384	57	65.4	8.0	This study
*Rathayibacter rathayi*	1	–	–	–	69.3	<0.0625	Davis et al., 2018
*Rathayibacter tritici*	2	–	–	–	69.5	<0.0625	NZ_CP015515; PRJNA429011
*Streptomyces chartreusis*	1	14	11,988	65	70.8	nd	Doroghazi et al., 2011
*Streptomyces clavuligerus*	1	14	12,056	67	72.3	nd	Song et al., 2010
*Actinosynnema mirum*	1	12	10,993	75	73.7	nd	Land et al., 2009

*^*a*^The number of putative genes present in the identified tunicaminyluracil gene clusters. ^*b*^The TGC size is based on the reference strains, *R. toxicus* FH79 (Sechler et al., 2017), *R. iranicus* FH6, *R. agropyri* CA4 (Schroeder et al., 2018), *R. woodii* FH236, *S. chartreusis* NRRL 3882 (Doroghazi et al., 2011), *S. clavuligerus* NRRL 3585 (Song et al., 2010), and *A. mirum* DSM 43827 (Land et al., 2009). ^*c*^The tunicamycin-minimum inhibitory concentration (MIC) values for evaluated *Rathayibacter* species. ^*d*^See Figure 3 for details. −, Not applicable; nd, not determined.*

### Genome Sequencing and Assembly

Three sequencing platforms, 454 Junior (Roche, Basel, Switzerland), Illumina (Illumina, San Diego, CA, United States), and PacBio RSII (Pacific Biosciences, Menlo Park, CA, United States), were used to sequence *Rathayibacter* strains (Supplementary Table S1). For *R. woodii* FH236, *R. iranicus* FH6, and *R. iranicus* FH177, a shotgun DNA library was constructed for the 454 Junior according to the manufacturer’s instructions, and three sequencing runs were performed. An additional PacBio sequencing library was prepared for *R. iranicus* FH6 and sequenced by the Genomics Lab at Washington State University. NGS DNA PCR-free libraries for *R. agropyri* strains and *R. iranicus* FH164 and FH176 were prepared according to the manufacturer’s protocols and sequenced on an Illumina MiSeq by the Georgia Genomics and Bioinformatics Core at the University of Georgia. Sequence data was assembled using Lasergene Ngen v12.0 (DNAStar), PATRIC v3.5.41 (Wattam et al., 2017), or Pacific Bioscience’s Hierarchical Genome-Assembly Process, as previously described (Savory et al., 2017; Sechler et al., 2017; Davis et al., 2018).

### Gene Annotation, Sequence Analysis, and Detection

The putative tunicaminyluracil gene clusters were identified and manually annotated using a combination of programs, including antiSMASH (Weber et al., 2015), BLAST search tools (default settings with *R. toxicus* TGC queries) (Altschul et al., 1997), InterPro (Finn et al., 2017), and Artemis v17.0.1 (Rutherford et al., 2000). Sequence conservation between the *Rathayibacter* species tunicaminyluracil-related gene clusters was analyzed using the default TBLASTX settings with the Artemis Comparison Tool v17.0.1 (Rutherford et al., 2000; Carver et al., 2005). The tunicaminyluracil gene cluster schematics were constructed and annotated using Easyfig v2.2.4 (Sullivan et al., 2011). Percent amino acid identity relative to *R. toxicus* FH79 was calculated using Clustal Ω (Clustal2.1) at EMBL-EBI (Sievers et al., 2011; Li et al., 2015). Pairwise average nucleotide identities (ANI) were calculated using autoANI and methods previously described (Goris et al., 2007; Davis et al., 2016, 2018). Publicly available tunicaminyluracil gene cluster sequences for *Rathayibacter* and related Actinobacteria species were downloaded from NCBI in October of 2017 (Land et al., 2009; Song et al., 2010; Doroghazi et al., 2011; Sechler et al., 2017; Davis et al., 2018).

Actinobacteria sequences for the conserved reference genes (*gyrB, dnaB, rpoB, recA, atpD*, and 16S RNA genes) were identified based on protein or DNA homology to genes of *R. toxicus*. The full length DNA sequences were concatenated and aligned with Muscle in MEGA v7.0 (Kumar et al., 2016). A maximum likelihood phylogeny was estimated using W-IQ-TREE (Trifinopoulos et al., 2016) using the best-fit substitution model with branch support being assessed with the Shimodaira-Hasegawa-like approximate likelihood ratio (SH-aLRT) (Guindon et al., 2010) and Ultrafast Bootstrap Approximation tests (UFB) (Hoang et al., 2018) using 1000 bootstrap replicates for each method. Trees were visualized using FigTree v1.4.4^1^. The same approach was used to construct phylogenetic trees of the putative tunicaminyluracil-associated genes (*tunA-tunF, tunH-tunL*).

MEGA v7.0, using Muscle and Clustal alignment default features, was used to assess the genetic diversity observed within the TGCs of *R. toxicus*, *R. iranicus*, and *R. agropyri* (Kumar et al., 2016). The predicted effects of missense mutations, among the TGC-conserved *Rathayibacter* genes (*tunA-tunL*), were predicted *in silico* with PROVEAN (Protein Variation Effect Analyzer) (Choi and Chan, 2015). If a missense mutation was predicted by PROVEAN to be deleterious, the effect of the mutation was analyzed using the program SIFT (Sorting Intolerant From Tolerant) (Vaser et al., 2016). The database for SIFT analyses consisted of amino acid sequences from *Streptomyces chartreusis* NRRL 3882, *S. clavuligerus* NRRL 3585, *Actinosynnema mirum* DSM 43827, *R. toxicus* FH79, *R. toxicus* FH232, and *R. woodii* FH236. A missense mutation was designated deleterious only if both algorithms predicted a deleterious mutation.

PCR was used to test for the presence of a TGC in strains of *R. toxicus, R. iranicus*, and *R. woodii* without associated genome sequences. Primers designed to amplify *tunA, tunC, tunF* were used (Supplementary Table S4). Amplification using EmeraldAmp MAX HS PCR Master Mix (Takara Bio Inc., Otsu, Shiga, Japan) or PrimeSTAR GXL DNA polymerase (Takara Bio Inc., Otsu, Shiga, Japan) was performed in an Applied Biosystems GeneAmp 9700 thermal cycler (Thermo Fisher Scientific, Waltham, MA, United States). Approximately 30 ng of extracted total DNA was used per 25 μL reaction with 0.2 μM of each primer. PCR using EmeraldAmp was performed with the following parameters: initial preheat for 2 min at 98°C; 32 cycles at 98°C for 10 s, a primer-pair-specific temperature for 20 s (Supplementary Table S4), and 72°C for 90 s; a final extension at 72°C for 2 min; and held at 10°C. PCR using PrimeSTAR was performed as a two-step reaction with 32 cycles at 98°C for 10 s and 75°C for 10 min. PCR products were electrophoresed on 1% GelGreen-stained 0.5x TAE agarose gels at 80 V for 45–60 min.

### Tunicamycin Sensitivity Assays

Differences in tunicamycin sensitivity were assessed with *Streptomyces-*produced tunicamycin (Sigma-Aldrich, St. Louis, MO, United States). Standardized methods for determining the minimum inhibitory concentration for tunicamycin were performed (Andrews and Andrews, 2001). Independent batches of tunicamycin were dissolved in alkaline water (pH > 9.0) and prepared as twofold dilutions in 6-well Falcon tissue culture plates (Corning Inc., Corning, NY, United States) containing 3 mL of modified YGM media. Wells were inoculated with 3 μL (1:1000) of a fresh *Rathayibacter* culture (OD_600_ < 1.0) and incubated on a rotary shaker at 28°C. Each plate contained positive (no tunicamycin) and negative (media only) control wells. Atoxigenic *Rathayibacter* species consisted of four strains of *R. rathayi* and three strains of *R. tritici*. Toxigenic *Rathayibacter* species consisted of four strains of *R. iranicus*, three strains of *R. toxicus*, 12 strains of *R. agropyri*, and two strains of *R. woodii.* Growth was observed visually at 7- and 14-days post inoculation. The entire experiment was repeated twice.

## Results

To determine if the TGC is unique to *R. toxicus*, we analyzed both previously and newly sequenced *Rathayibacter* genomes (Supplementary Table S1). Twenty-one globally collected *Rathayibacter* strains were previously sequenced, representing 18 strains of *R. toxicus*, 2 strains of *R. iranicus*, and one strain of *R. agropyri* (Sechler et al., 2017; Davis et al., 2018). To complement the previously sequenced genomes, a single isolate of *R. woodii*, 11 *R. agropyri* strains, and an additional four *R. iranicus* strains were sequenced (Supplementary Table S1) (Davis et al., 2018).

### *Rathayibacter woodii* Represents a New Species Group in the *Rathayibacter* Genus

**FIGURE 1:**
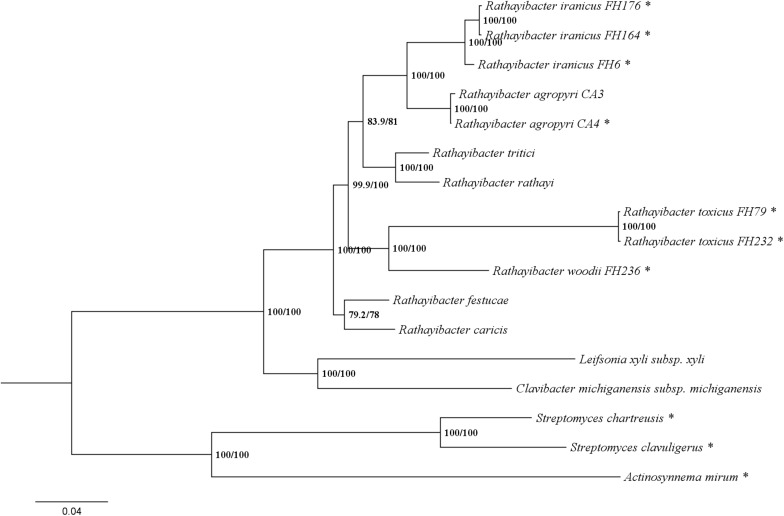
*Rathayibacter woodii* is a distinct species in the *Rathayibacter* genus. The phylogeny is based on concatenated *gyrB*, *dnaB*, *rpoB*, *recA*, *atpD*, and 16S rDNA nucleotide sequences from *Rathayibacter* and related Actinobacteria. The percentage of replicate trees in which the associated taxa clustered together are shown at the nodes: IQ-TREE SH-aLRT bootstrap support/Ultrafast bootstrap support. Actinobacteria with putative tunicaminyluracil gene clusters are designated with an asterisk.

Previous chemotaxonomic reports suggested that *R. woodii* is a distinct *Rathayibacter* species (Riley et al., 2004). Results from an Average Nucleotide Identity comparison and maximum likelihood (ML) phylogenetic analysis were consistent with the previous chemotaxonomic reports (Figure 1 and Supplementary Table S2). The phylogenetic analysis based on six conserved loci had similar topology to the whole-genome ML tree recently constructed for the *Rathayibacter* genera (Davis et al., 2018). All tested *Rathayibacter* species cluster together and clearly separate from the closely related genus *Leifsonia* and related Actinobacteria (Figure 1). Within the *Rathayibacter* clade, *R. woodii* is on a separate branch and appears most genetically similar to *R. toxicus*, as determined on the basis of strong bootstrap support (100%) (Figure 1).

### Three Species of *Rathayibacter* Have Novel Tunicaminyluracil Biosynthetic Gene Clusters

**FIGURE 2:**
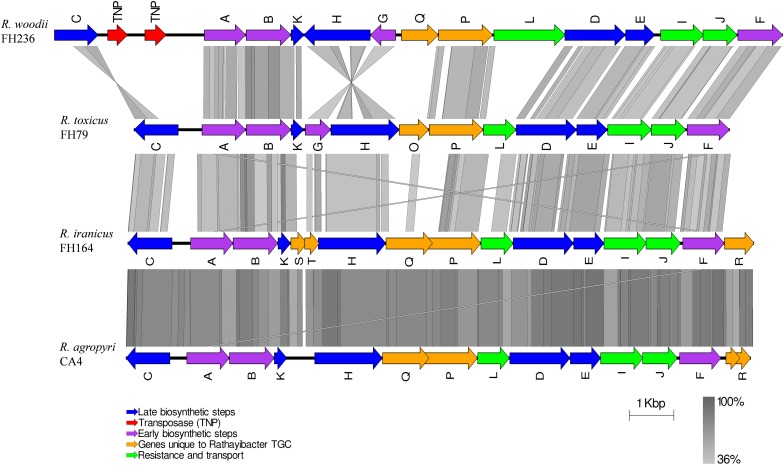
Tunicaminyluracil-related gene cluster comparisons of *Rathayibacter* species. Microsynteny and sequence conservation between the tunicaminyluracil-related gene clusters for *Rathayibacter toxicus* FH79 (Sechler et al., 2017), *R. iranicus* FH164, *R. woodii* FH236, and *R. agropyri* CA4 (Schroeder et al., 2018). Predicted ORFs are shown in their respective orientation with alignment blocks corresponding to the level of sequence identity.

The *R. toxicus* TGC contains 14 genes, of which 13 genes (all except *tunC*) are predicted to comprise a single polycistronic operon (Figure 2) (Sechler et al., 2017). These TGC-associated genes and those of *S. chartreusis* were used as queries to search for homologs of tunicaminyluracil-related genes in the newly sequenced *Rathayibacter* genomes. Three tunicaminyluracil-related biosynthetic gene clusters were identified in *R. iranicus, R. woodii*, and *R. agropyri*. Most genes necessary for tunicaminyluracil biosynthesis were conserved among the four *Rathayibacter* species, *Streptomyces* species, and *Actinosynnema mirum*, and unique genes were also present (Tables 1, 2). The TGCs of the four *Rathayibacter* species are similar in having lower GC-content relative to their respective genomes and having homologous genes essential for tunicaminyluracil biosynthesis (Table 1). No homologs of essential tunicaminyluracil-related genes were identified in other sequenced *Rathayibacter* species.

**TABLE 2:** Genes associated with the putative tunicaminyluracil-related gene clusters of *Rathayibacter toxicus* FH79 (Sechler et al., 2017), *R. agropyri* CA4 (Schroeder et al., 2018), *R. iranicus* FH164, *R. woodii* FH236, *Streptomyces chartreusis* 3882 (Doroghazi et al., 2011), and *Actinosynnema mirum* DSM 43827 (Land et al., 2009).

		**Protein length (aa) and% amino acid identity relative to *R. toxicus* FH79^b^**					
**Gene^a^**	**Description**	***R. toxicus***	***R. agropyri***	***R. iranicus***	***R. woodii***	***S. chartreusis***	***A. mirum***
***tunA***	**Epimerase**	330	320(54%)	320(53%)	313(46%)	321(42%)	322(38%)
***tunB***	**Radical SAM**	337	337(86%)	337(84%)	338(71%)	338(68%)	340(67%)
***tunC***	**Acetyltransferase**	333	331(52%)	333(51%)	330(38%)	318(28%)	318(29%)
***tunD***	**Glycosyltransferase**	454	453(55%)	453(55%)	456(40%)	472(37%)	451(38%)
***tunE***	***N*-deacetylase**	232	230(62%)	230(62%)	222(49%)	234(42%)	230(43%)
*tunF*	Epimerase	320	312(60%)	312(60%)	338(45%)	327(35%)	328(35%)
*tunG*	Histidine phosphatase	192	−	−	192(36%)	203(31%)	223(30%)
***tunH***	**Nucleotide pyrophosphatase**	515	509(50%)	509(48%)	499(39%)	515(38%)	510(37%)
***tunI***	**ABC transporter**	330	320(49%)	320(50%)	324(34%)	304(32%)	302(29%)
***tunJ***	**ABC-2 transporter**	262	261(65%)	261(64%)	260(47%)	262(44%)	253(47%)
*tunK*	Acyl carrier protein	96	96(45%)	96(44%)	98(33%)	81(30%)	85(28%)
*tunL*	Phospholipid phosphatase	247	239(32%)	243(31%)	537(22%)	229(22%)	293(23%)
*tunM*	Methyltransferase	−	−	−	−	216	−
*tunN*	Pyrophosphatase	−	−	−	−	152	−
*tunO*	Hypothetical protein	224	−	−	−	−	−
*tunP*	Polyketide synthase	407	408(57%)	407(58%)	408(48%)	−	−
*tunQ*	Enoyl-CoA hydratase/isomerase	−	351	351	279	−	−
*tunR*	4′-phosphopantetheinyl transferase	−	224	224	−	−	−
*tunS*	Hypothetical protein	−	−	122	−	−	−
*tunT*	Hypothetical protein	−	−	111	−	−	−

*^*a*^Genes in bold text are essential for tunicamycin biosynthesis and immunity in *Streptomyces chartreusis* (Widdick et al., 2018). ^*b*^–, Absence of gene.*

The TGCs of the other three species vary in gene order, direction, and total gene count relative to that of *R. toxicus* (Tables 1, 2 and Figure 2). The *R. woodii-*TGC is larger (∼16 kb in length) than the *R. toxicus-*TGC, likely a consequence of the insertion of transposase genes between an inverted *tunC* and the rest of the locus. *R. woodii* has homologs of the 13 genes present in the TGC of *R. toxicus* (*tunA-tunL, tunP)*; the hypothetical *R. toxicus* gene *tunO* is replaced by an enoyl-CoA hydratase/isomerase gene (*tunQ*). The phospholipid phosphatase-encoding gene (*tunL*) of *R. woodii* is approximately double the length of *tunL* homologs in other *Rathayibacter* species and its translated sequence shares 30% amino acid identity to the C-terminal region of the *R. toxicus* TunL; however, the N-terminal region (∼318 aa) lacks any conserved motifs (Table 2). In *R. woodii* there is an inversion of *tunG* and *tunH*, interrupting a locus that would otherwise have the potential to encode a single polycistronic message (Figure 2).

*Rathayibacter iranicus* possesses a similarly sized TGC (∼14 kb in length) to *R. toxicus* and has 16 possible genes (Table 1). Three of the hypothetical open reading frames (ORFs), *tunS, tunT*, and *tunR*, appear to be unique. However, TunT appears to be a truncated C-terminal variant of TunG, as it shares 47% amino acid identity. *R. iranicus* lacks *tunG.* TunR is predicted to encode a 4′-phosphopantetheinyl transferase and its gene (*tunR*) is located at the end of the locus.

**FIGURE 3:**
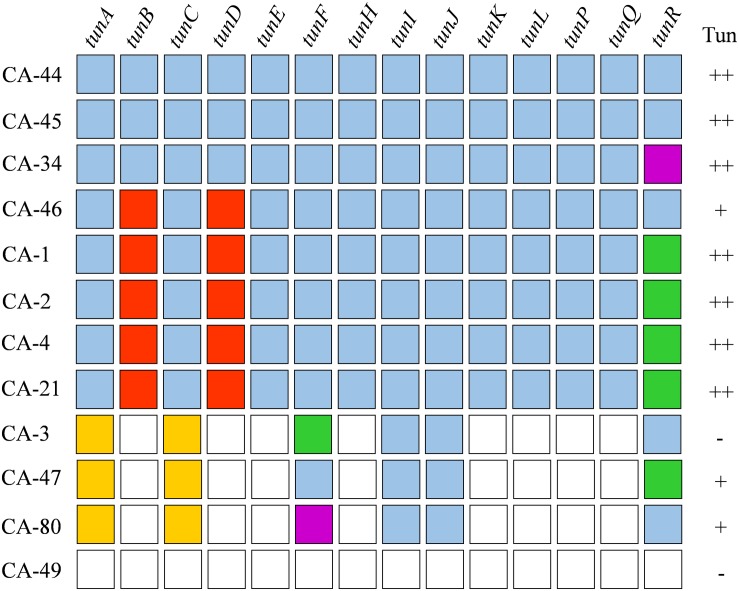
Genetic variability of tunicaminyluracil-related gene clusters between *Rathayibacter agropyri* strains. Tunicaminyluracil-related genes are listed across the top, *R. agropyri* strains are listed on the left side, and tunicamycin tolerance is denoted in the right most column. *Rathayibacter agropyri* tunicaminyluracil-associated genes are displayed in alphabetical order: gene is present (blue), gene is absent (white), gene is partially present (gold), a frameshift mutation is present (green), a mutation is predicted to affect protein function (red), and a transposon insertion (purple). Tunicamycin tolerance was assessed where strains showed no resistance (–), growth at 1 μg/ml (+), or growth above 1 μg/ml (++).

The TGC of *R. agropyri* is most similar in gene sequence, composition, and order as the TGC of *R. iranicus* (Figure 2). *R. agropyri* lacks the histidine phosphatase gene (*tunG*) and possesses the 4′-phosphopantetheinyl transferase (*tunR*) gene at the terminal end of the locus. However, *R. agropyri* contrasts with other TGC-containing *Rathayibacter* species, in exhibiting a high degree of presence/absence polymorphisms of the TGC within the taxon. Eight strains are predicted to have complete TGCs. However, we could not identify a TGC locus in the genome sequence of *R. agropyri* strain CA-49. Last, in strains CA-3, CA-47, CA-80, we could detect only incomplete TGCs that circumscribe complete coding sequences for *tunI, tunJ, tunF*, and *tunR* as well as fragmented sequences of *tunA* and *tunC* (Figure 3).

### Independent Horizontal Gene Transfer of the *R. woodii* TGC

**FIGURE 4:**
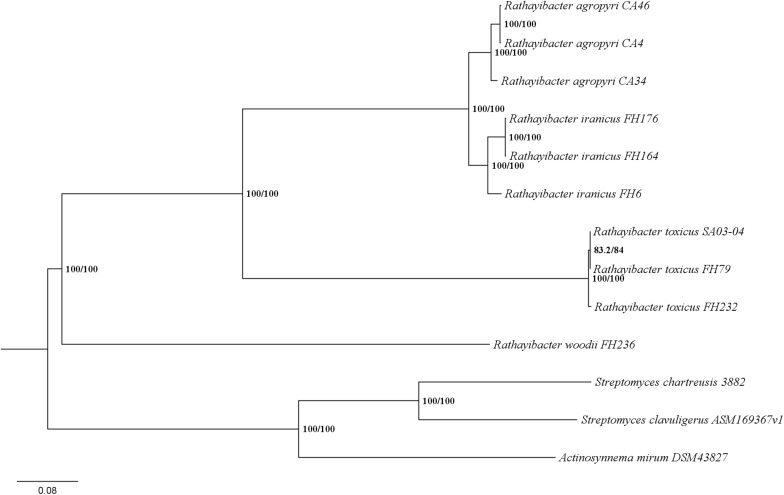
The tunicaminyluracil-related gene cluster of *Rathayibacter woodii* is distinct from associated Actinobacteria. The phylogeny is based on concatenated tunicaminyluracil-related genes (*tunA-tunL*), with the exception of *tunG* due to the lack of conservation between species. The percentage of replicate trees in which the associated taxa clustered together are shown at the nodes: IQ-TREE SH-aLRT bootstrap support/Ultrafast bootstrap support.

Despite the genetic relatedness of *R. woodii* to *R. toxicus* (Figure 1), the TGC of *R. woodii* is different from those of *R. toxicus*, *R. iranicus*, and *R. agropyri* (Figure 2). A phylogenetic analysis based on the concatenation of eleven TGC-conserved genes placed the TGCs from *R. toxicus*, *R. iranicus*, and *R. agropyri* into a clade with bootstrap support of 100% (Figure 4). The Eurasian *R. iranicus* isolates and the North American *R. agropyri* isolates appear to have highly conserved TGCs. However, the TGC of *R. woodii* is on a branch separate from *R. toxicus*, *R. iranicus*, *R. agropyri*, and all other characterized TGC-possessing Actinobacteria (Figure 4). The phylogeny of the conserved TGC is incongruent with the *Rathayibacter* genera phylogenetic tree, suggesting independent horizontal gene transfer of the *R. woodii* TGC. Further evidence of horizontal TGC transfers is the combination of a low GC content, relative to the genome, and flanking transposase genes (Table 1). Except for *R. toxicus*, all TGC-possessing *Rathayibacter* species have transposase-encoding genes downstream of *tunC*; and the recently isolated *R. agropyri* strain, CA-34, has upstream and downstream transposase genes flanking the TGC.

### Presence of Rare Regulatory Codons in the *Rathayibacter* TGC

The overrepresentation of rare TTA leucine codons in *Rathayibacter* TGCs, relative to the rest of the genome, could be indicative of past horizontal gene transfer events and post-translational regulation, as in other Actinobacteria (Chandra and Chater, 2008; Bedhomme et al., 2019) (Table 3). Approximately 2.4% of *R. toxicus* leucine codons are represented by the rare TTA codon in the genome; however, within the TGC, the rare TTA codon represents 11% of all sites coding for leucine (Table 3). Similarly, *R. iranicus*, *R. woodii*, and *R. agropyri* have <1% of rare TTA codons represented in their genomes, but between 4.2–5.7% within their respective TGCs. In contrast, *Streptomyces* species possess a similar percentage of rare TTA codons (<1%) in both the TGC and genome (Table 3).

**TABLE 3:** The overrepresentation of rare TTA leucine codons within the tunicaminyluracil biosynthetic gene clusters of putatively toxigenic *Rathayibacter* and *Streptomyces* species.

	**Number of rare TTA codons (% of total Leu)^∗^**					
**Gene**	***R. toxicus* FH79**	***R. iranicus* FH6**	***R. woodii* FH236**	***R. agropyri* CA34**	***S. clavuligerus* 3585**	***S. chartreusis* 3882**
*tunA*	3 (13.6)	2 (7.4)	1 (3.2)	1 (3.8)	0 (0.0)	3 (8.8)
*tunB*	6 (16.7)	2 (5.4)	0 (0.0)	3 (8.1)	0 (0.0)	0 (0.0)
*tunC*	4 (13.8)	3 (10.0)	0 (0.0)	3 (10.7)	0 (0.0)	0 (0.0)
*tunD*	6 (11.5)	2 (4.0)	3 (5.4)	2 (3.9)	0 (0.0)	0 (0.0)
*tunE*	2 (9.1)	0 (0.0)	1 (7.7)	0 (0.0)	0 (0.0)	0 (0.0)
*tunF*	2 (9.5)	1 (3.6)	0 (0.0)	1 (3.7)	0 (0.0)	0 (0.0)
*tunG*	1 (5.9)	–	2 (10.5)	–	0 (0.0)	0 (0.0)
*tunH*	6 (12.5)	3 (6.5)	2 (3.9)	4 (8.3)	0 (0.0)	0 (0.0)
*tunI*	4 (11.4)	0 (0.0)	3 (7.5)	1 (2.6)	0 (0.0)	0 (0.0)
*tunJ*	4 (10.3)	1 (2.6)	0 (0.0)	2 (5.1)	0 (0.0)	0 (0.0)
*tunK*	0 (0.0)	0 (0.0)	1 (9.1)	0 (0.0)	0 (0.0)	0 (0.0)
*tunL*	2 (11.1)	2 (8.0)	3 (4.7)	2 (8.0)	0 (0.0)	0 (0.0)
*tunM*	–	–	–	–	1 (3.6)	1 (4.0)
*tunN*	–	–	–	–	0 (0.0)	0 (0.0)
*tunO*	1 (5.6)	–	–	–	–	–
*tunP*	3 (12.0)	0 (0.0)	1 (2.9)	0 (0.0)	–	–
*tunQ*	–	3 (8.3)	3 (8.3)	5 (13.2)	–	–
*tunR*	–	0 (0.0)	–	–	–	–
*tunS*	–	1 (12.5)	–	–	–	–
*tunT*	–	0 (0.0)	–	–	–	–
TGC Total	44 (11.2)	20 (4.5)	20 (4.2)	24 (5.7)	1 (0.2)	4 (0.9)
Genome Total	1619 (2.4)	457 (0.5)	593 (0.7)	359 (0.4)	172 (0.1)	Not sequenced

*^∗^−, absence of gene.*

### Non-synonymous Mutations Within the TGCs of *R. toxicus* and *R. iranicus* Are Not Predicted to Impact Protein Function

The TGC is present in all 37 strains of *R. toxicus* and 23 strains of *R. iranicus* (Supplementary Table S1). Sequence analysis of the *R. toxicus* TGC revealed a total of 66 single nucleotide polymorphisms (SNPs) present within the tunicaminyluracil-associated genes, relative to FH79. A total of three *R. toxicus*-TGC haplotypes were identified between the 26 sequenced strains with most SNPs belonging to the TGC of *R. toxicus* FH100/232 (Table 4). Only 2 SNPs (*tunB* and *tunD*) differentiated the emerging *R. toxicus* population, SA03-04, from the type strain, *R. toxicus* FH79. Comparing the dominant *R. toxicus* TGC haplotype to *R. toxicus* FH100/232 revealed numerous polymorphisms, non-synonymous mutations, and insertions/deletions (INDELs) (Table 4). A six nucleotide INDEL was present at the 3′ end of *tunD*, resulting in a premature stop codon and subsequent truncation by two amino acids. The INDEL further modified the downstream gene *tunE* via the addition of two amino acids to the N-terminus (Table 4). Within *R. toxicus*, *tunA* was the most polymorphic gene with 21 SNPs corresponding to 2.1% variation. No SNPs were observed in *tunF, tunJ*, and *tunK.* With the exception of the INDEL, all remaining 35 non-synonymous mutations resulted in missense mutations that were predicted *in silico* by PROVEAN software to not affect protein function (Table 4) (Choi and Chan, 2015).

**TABLE 4:** Genetic variation of the tunicaminyluracil biosynthetic gene cluster in sequenced *Rathayibacter toxicus, R. iranicus*, and *R. agropyri* strains, and their predicted impact to protein function.

**Gene**	**Gene size (bp)^a^**	**No. of strains**	**No. of haplotypes**	**No. (%) of polymorphic sites**	**No. of INDELs (bp)^b^**	**No. of non-synonymous mutations^c^**	**Type of non-synonymous mutation**	**Predicted mutation tolerated^d^**
*R. toxicus*								
*tunA*	993	26	2	21(2.1%)	–	8	Missense	Neutral
*tunB*	1014	26	3	4(0.4%)	–	1	Missense	Neutral
*tunC*	1002	26	2	8(0.8%)	–	2	Missense	Neutral
*tunD*	1365	26	3	5(0.4%)	1 (6 bp)	3	Nonsense/Missense	Neutral
*tunE*	699	26	2	11(1.6%)	1 (6 bp)	4	Missense	Neutral
*tunF*	963	26	2	0(0.0%)	–	0	–	–
*tunG*	579	26	2	1(0.2%)	–	0	–	–
*tunH*	1548	26	2	6(0.4%)	–	4	Missense	Neutral
*tunI*	993	26	2	6(0.6%)	–	5	Missense	Neutral
*tunJ*	789	26	2	0(0.0%)	–	0	–	–
*tunK*	291	26	2	0(0.0%)	–	0	–	–
*tunL*	744	26	2	1(0.1%)	–	1	Missense	Neutral
*tunO*	675	26	2	10(1.5%)	–	6	Missense	nd
*tunP*	1224	26	2	1(0.1%)	–	1	Missense	nd
*R. iranicus*								
*tunA*	978	6	2	41(4.3%)	1 (15 bp)	12	Missense	Neutral
*tunB*	1014	6	3	42(4.1%)	–	12	Missense	Neutral^∗^
*tunC*	1002	6	3	54(5.4%)	–	20	Missense	Neutral
*tunD*	1362	6	3	51(3.7%)	–	20	Missense	Neutral
*tunE*	693	6	3	16(2.3%)	–	2	Missense	Neutral
*tunF*	939	6	3	30(3.2%)	–	11	Missense	Neutral
*tunH*	1530	6	3	76(5.0%)	–	36	Missense	Neutral
*tunI*	963	6	4	31(3.2%)	–	14	Missense	Neutral
*tunJ*	786	6	3	27(3.4%)	–	7	Missense	Neutral
*tunK*	291	6	2	24(8.2%)	–	11	Missense	Neutral
*tunL*	732	6	3	25(3.4%)	–	15	Missense	Neutral
*tunP*	1224	6	3	32(2.6%)	–	8	Missense	nd
*tunQ*	1056	6	2	31(2.9%)	–	15	Missense	nd
*tunR*	675	6	3	20(3.0%)	–	11	Missense	nd
*tunS*	369	6	2	41(11.1%)	–	26	Missense	nd
*tunT*	213	6	2	5(2.3%)	1 (3 bp)	3	Nonsense/Missense	nd
*R. agropyri*								
*tunA*	963	8	5	23(2.4%)	–	7	Missense	Neutral
*tunB*	1014	8	4	25(2.5%)	–	7	Missense	Deleterious
*tunC*	996	8	4	21(2.1%)	–	7	Missense	Neutral
*tunD*	1362	8	6	29(2.1%)	–	13	Missense	Deleterious
*tunE*	693	8	6	14(2.0%)	–	4	Missense	Neutral
*tunF*	939	8	5	25(2.7%)	–	13	Missense	Neutral
*tunH*	1530	8	5	52(3.4%)	–	22	Missense	Neutral^∗^
*tunI*	963	8	6	38(3.9%)	–	15	Missense	Neutral
*tunJ*	786	8	4	16(2.0%)	–	3	Missense	Neutral
*tunK*	291	8	4	8(2.7%)	–	5	Missense	Neutral
*tunL*	720	8	5	15(2.1%)	1 (3 bp)	8	Missense	Neutral
*tunP*	1227	8	6	22(1.8%)	–	9	Missense	nd
*tunQ*	1056	8	6	24(2.3%)	–	12	Missense	nd
*tunR*	675	8	6	50(7.4%)	2 (1 bp)	33	Nonsense/Missense	nd

*^*a*^The gene size based on the reference strains *R. toxicus* FH79 (Sechler et al., 2017), *R. iranicus* FH6, and *R. agropyri* CA4 (Schroeder et al., 2018). ^*b*^The number of unique INDELs (insertion/deletion) within sequenced fragments. Each INDEL was treated as a single polymorphic site. ^*c*^The number of non-synonymous mutations does not include INDELs. ^*d*^The functional effect of missense mutations, among the TGC-conserved *Rathayibacter* genes (*tunA-tunL*), were predicted *in silico* with PROVEAN (Protein Variation Effect Analyzer) and SIFT (Sorting Intolerant From Tolerant) (Choi and Chan, 2015; Vaser et al., 2016). ^∗^, a missense mutation was predicted to be deleterious with PROVEAN, but tolerated with SIFT; nd, not determined due to the lack of adequate reference sequences.*

Sequence data from six diverse *R. iranicus* strains collected from Iran and Turkey revealed five unique TGC haplotypes, corresponding to approximately 600 SNPs (Table 4). The TGC of *R. iranicus* type strain FH6 differed from *R. iranicus* FH154, FH157, and FH177 by only 1-2 SNPs; however, most TGC-associated SNPs were attributed to *R. iranicus* strains FH164 and FH176. A 15 nucleotide INDEL at the beginning of *tunA* was identified in both *R. iranicus* FH164 and FH176 and is predicted to result in a truncated protein (Table 4). *R. iranicus* FH6, FH154, FH157, and FH177 each have a premature stop codon, predicted to truncate TunT 22 amino acids from the start position. An additional three nucleotide INDEL in *tunT* is present in *R. iranicus* FH164 and FH176, resulting in the deletion of a codon for tryptophan (W77). The sequence diversity observed in the TGC of *R. iranicus* FH164 and FH176 were mirrored in the rest of the genome, consistently separating *R. iranicus* FH164 and FH176 from the *R. iranicus* type strain FH6 (Figures 1, 4). A total of 223 non-synonymous mutations were identified within the *R. iranicus* TGC, but the missense mutations were predicted to be tolerated on the basis of *in silico* analysis (Table 4) (Choi and Chan, 2015; Vaser et al., 2016). A single amino acid substitution in TunB (I48T) of *R. iranicus* FH164 and FH176 was predicted by PROVEAN, but not SIFT, to affect protein function (Table 4).

### Genetic Variability Within *R. agropyri* Has Resulted in Strains Having Complete, Partial, or No TGC

Sequencing an additional 11 *R. agropyri* strains, collected from the Western United States, revealed eight strains with a complete TGC, three strains with TGC remnants, and a single strain (CA-49) that had no evidence of a TGC (Figure 3). The presence of a TGC does not appear to be spatially or temporally correlated, with *R. agropyri* strains collected between 1945 and 2013 possessing complete TGCs (Figure 3 and Supplementary Table S1). Among the eight strains that possess a TGC, a total of 6 unique haplotypes were identified with 359 SNPs and a single 901 bp transposase in *tunR* of strain CA-34. The 4’-phosphopantetheinyl transferase (*tunR*) gene has the highest percentage of mutations, which resulted in frame shifts, nonsense mutations, or INDELS in five of the eight *R. agropyri* strains (Table 4 and Figure 3). A total of 158 non-synonymous mutations were identified within the *R. agropyri* TGC with most missense mutations predicted to be tolerated *in silico* (Choi and Chan, 2015; Vaser et al., 2016). However, the single amino acid substitutions in TunB (D282Y) and TunD (G30R) of *R. agropyri* strains CA-1, CA-2, CA-4, CA-21, and CA-46 were predicted to affect protein function in both PROVEAN and SIFT analyses (Table 4 and Figure 3). An additional amino acid substitution in TunH (R247C) of *R. agropyri* strains CA-1, CA-2, CA-4, CA-21, and CA-46 was predicted by PROVEAN, but not SIFT, to affect protein function (Table 4).

The four *R. agropyri* strains that either lacked or had remnants of a TGC were isolated from samples collected between 1950 and 2014 from Idaho, Colorado, or Washington (Supplementary Table S1). Complete or partial TGCs were in the same chromosomal position with flanking ORFs, except for strain CA-49, which lacked a TGC. *Rathayibacter agropyri* strains CA-3, CA-47, and CA-80 had complete *tunI* and *tunJ* ORFs and approximately 5 and 25 amino acids of the N-terminus of TunA and TunC, respectively. *tunF* was complete in CA-47 but contained nonsense mutations and transposon insertions in CA-3 and CA-80, respectively. *tunR* was complete in CA-3 and CA-80, while CA-47 contained an early nonsense mutation.

### *Rathayibacter* Strains With a TGC Locus Are Tolerant to Exogenous Tunicamycin

Tolerance by atoxigenic and toxigenic *Rathayibacter* species to tunicamycin was evaluated to determine if the presence or absence of the TGC influenced tunicamycin sensitivity. Atoxigenic strains of *R. rathayi* and *R. tritici* were highly sensitive to externally applied tunicamycin with a minimum inhibitory concentration (MIC) value < 0.0625 μg/mL (Table 1 and Supplementary Table S3). In contrast, putatively toxigenic *R. toxicus*, *R. iranicus*, and *R. woodii*, were each tolerant, with MIC values of 8.0 μg/mL to externally applied tunicamycin (Table 1 and Supplementary Table S3). *R. agropyri* strains showed a range of sensitivity from <0.0625 to 8.0 μg/mL with sensitivity correlated to the absence of a complete TGC. No bacterial growth was observed in the negative control wells.

## Discussion

The distribution and conservation of tunicaminyluracil gene clusters within the nematode-vectored, grass-associated *Rathayibacter* genera has not been previously investigated; and tunicaminyluracil biosynthesis has been reported in only a handful of soil-associated bacterial species (Doroghazi et al., 2011). Therefore, genomic analyses were performed on available toxigenic and atoxigenic *Rathayibacter* species isolated from diverse geographic locations to determine if putative TGCs were conserved in this plant pathogenic taxon. In this study, we identified novel tunicaminyluracil-related gene clusters in three suspected toxigenic *Rathayibacter* species that infect wheat and grasses.

The first confirmed appearance of *R. toxicus*-like poisoning outside of Australia was documented in the Western Cape Province of South Africa in 1980 (Schneider, 1981). Schneider (1981) identified a *Rathayibacter* species in contaminated fodder that was responsible for livestock poisonings and quickly presumed it to be *R. toxicus*, although the identity of the bacterial species was never confirmed. Based on our results, it is plausible that the South African livestock poisonings were caused by an endemic toxigenic *Rathayibacter* species, such as *R. woodii*, as opposed to a foreign *R. toxicus* strain. Numerous nematode and *R. woodii* bacterial galls were isolated in the same Western Cape Province where the previous suspected *R. toxicus*–like poisonings occurred (Schneider, 1981; Riley and Swart, 2004; Grewar et al., 2009). *R. woodii* was isolated from dune grass (*Ehrharta villosa* var. *villosa*) in association with the leaf gall nematode, *Anguina woodi* (Riley et al., 2004; Riley and Swart, 2004). In contrast, Schneider (1981) associated the isolated toxigenic *Rathayibacter* species with *Lolium* species seed and the seed gall nematode *Anguina agrostis*. However, numerous studies have demonstrated that *Rathayibacter* grass-host range is determined by the nematode vector and not the bacterium, with *R. toxicus* being able to colonize multiple grass and *Anguina* species (Edgar et al., 1982; Riley and McKay, 1990, 1991; Riley et al., 2001). Future research will be needed to evaluate the ability of *R. woodii* to attach to the cuticle of other *Anguina* species.

Genetically similar tunicaminyluracil-related gene clusters were also identified in the geographically separated populations of *R. iranicus* and *R. agropyri. R. iranicus* has only been identified in Iran and Turkey and causes a gumming disease of wheat similar to atoxigenic *R. tritici* (Bird, 1981; Postnikova et al., 2009; Fattah and Al-Assas, 2010). No supporting literature has described *R. toxicus*-like poisonings of livestock in Iran or Turkey, but wheat seed is rarely utilized as a food source for livestock. In contrast, historical accounts of livestock poisonings, associated with fodder contaminated with nematode galls, were documented in Oregon during the mid-twentieth century (Haag, 1943; Shaw and Muth, 1949; Galloway, 1961; Jensen, 1961).

The TGCs of *R. iranicus* and *R. agropyri* both lack the histidine phosphatase gene *tunG*. In place of *tunG, R. iranicus* has two hypothetical ORFs, with one of these putative genes (*tunT*) predicted to encode a truncated protein with homology to the C-terminal portion of TunG. However, the accumulation of mutations in *tunT* across diverse *R. iranicus* strains, and the lack of a conserved histidine phosphatase catalytic core, suggests that *tunT* may be a non-functional remnant of a histidine phosphatase. It remains unknown if *R. iranicus* or *R. agropyri* synthesize tunicaminyluracil, but Widdick et al. (2018) determined that just six genes (*tunABCDEH)* are essential for tunicamycin biosynthesis in *S. chartreusis*, with *tunI* and *tunJ* being required for immunity (Widdick et al., 2018). The remaining non-essential TGC-associated genes (*tunFGKL*) appear redundant with homologs involved in *S. chartreusis* primary metabolism, but may allow for proper stoichiometric mixtures at the initial stages of tunicamycin biosynthesis (Widdick et al., 2018).

Environmental factors that trigger the synthesis of tunicaminyluracil antibiotics in *Rathayibacter* species are unknown, and in-field production appears to be sporadic in the case of *R. toxicus* infections (Kowalski et al., 2007). The inconsistent production of tunicaminyluracil, both *in vitro* and *in vivo*, could be due to a variety of complex interactions, but it does not appear to be a result of *R. toxicus* strains lacking a functional TGC. The TGC was conserved in all strains of *R. toxicus*, *R. woodii*, and *R. iranicus*, and mutations were not predicted to disrupt protein function. In contrast, *R. agropyri* genotypes were highly diverse with strains having complete, partial, or missing TGCs. The accumulation of mutations predicted to impact protein function in TunB and TunD were also prevalent in *R. agropyri.* Of the 12 *R. agropyri* strains, only three strains, CA-44, CA-45, and CA-34, have all the essential genes (without mutations) necessary for tunicaminyluracil production. Interestingly, *R. agropyri* CA-45 was collected from Oregon in 1950 during the state’s reported *R. toxicus*-like poisoning events. Similarly, strains CA-44, CA-46, CA-2, CA-47, and CA-49 were collected in 1950 from nearby states, but either lacked or had diverse TGC loci. Recently collected *R. agropyri* isolates (CA-34, CA-21, and CA-80) possess TGCs that mirror the diversity observed in strains collected during the mid-20th century, with *R. agropyri* CA-34 possessing all essential tunicaminyluracil genes. The diversity of TGC sequences in *R. agropyri* genotypes could be one explanation for why widespread livestock toxicosis events are not reported in the United States as they are in Australia.

There are three *R. agropyri* strains with partial TGCs: CA-3, CA-47, and CA-80. The only two genes present in all three strains, *tunI* and *tunJ*, are the two required for immunity in *S. chartreusis* (Widdick et al., 2018). However, the requirements for full immunity to exogenous tunicamycin appear to be more complex in *R. agropyri*. None of these three strains grow at as high concentrations of tunicamycin as many of the strains with complete TGCs (Figure 3) and CA-3 exhibits no immunity to tunicamycin despite full-length copies of *tunI* and *tunJ*. Therfore, it is likely that additional genes, either in the TGC or elsewhere in the genome, contribute to full immunity in *R. agropyri.* Never the less, it is possible that the partial immunity given by *tunI* and *tunJ* confers a selective advantage to *R. agropyri*.

The low GC-content, overrepresentation of rare leucine codons, nearby transposase genes, and incongruence of phylogenetic trees strongly suggest that the TGC was horizontally acquired in the *Rathayibacter* genera, with likely at least two independent genetic transfer events. Although the origin of the *R. woodii* TGC remains unknown, it appears to be from a source that is different than the donor to *R. toxicus.* The TGC of *R. woodii* possesses the complete suite of tunicaminyluracil-related genes but is different in structure. Moreover, the putative TGC of *R. woodii* does not cluster with *R. toxicus*, despite the close genetic distance between the two species. While there are additional *Streptomyces* and *Actinosynnema* that contain TGCs closely related to the ones included in our analyses, there are no other putative TGCs in available bacterial genome sequences. Therefore, it is difficult to speculate on the origin of any of the *Rathayibacter* TGCs.

The presence of the TGC within multiple *Rathayibacter* species, the prevalence of the TGC locus among strains evaluated, the conservation and homology of tunicaminyluracil-essential genes, the increased tolerance to exogenous tunicamycin, and the historic reports of livestock poisonings, all suggest that these species possess(ed) functional tunicaminyluracil-related gene clusters. However, it is challenging to demonstrate that these *Rathayibacter* species synthesize biologically active tunicaminyluracil. The biological/environmental triggers that initiate tunicaminyluracil production *in vitro* are unknown and could differ among the species. Payne and Cockrum (1988) were intermittently able to induce *in vitro* production of tunicaminyluracil with *R. toxicus*, but required fresh field isolates, the process was highly strain specific, and tunicaminyluracil production was rapidly lost *in vitro* with successive subculturing (Payne and Cockrum, 1988). In fact, successful *in vitro* tunicaminyluracil production has only been consistently demonstrated within the *Streptomyces* genera, in which *Streptomyces* species are induced to synthesize tunicamycin under extended anaerobic conditions (Chen et al., 2010). Due to these limitations, tolerance to exogenous tunicamycin was used as a partial proxy for the TGCs functionality in *Rathayibacter* species. The genes required for tunicamycin immunity appear to be functional since TGC-containing *Rathayibacter* species were able to tolerate exogenous applications of tunicamycin, in contrast to atoxigenic species. Interestingly, the newly described TGCs do not appear to possess any regulatory genes, similar to *S. chartreusis* and *R. toxicus* (Wyszynski et al., 2010; Sechler et al., 2017). However, tunicaminyluracil production in *R. toxicus* (and other *Rathayibacter* species) may be translationally regulated through the utilization of rare TTA leucine codons, which are overrepresented in *Rathayibacter* TGCs, in a similar manner to antibiotic production in *Streptomyces* where _Leu_tRNA^UUA^ is temporally regulated and accumulates late in growth (Lawlor et al., 1987; Chater and Chandra, 2008). Unlike *R. toxicus* and the associated bacteriophage NCPPB3778, no bacteriophage have been implicated in toxin production by *R. iranicus*, *R. agropyri*, or *R. woodii* (Murray et al., 2017; Schneider et al., 2017).

The nematode vector *Anguina funesta*, and other *Anguina* species, are present in the United States (Meng et al., 2012). As discussed by Murray et al. (2017), the potential introduction of toxigenic *Rathayibacter* species, along with native *Anguina* species nematode populations, could have severe implications for United States agriculture. The widespread distribution and conservation of tunicaminyluracil-essential genes in diverse *Rathayibacter* species, collected from Australia, South Africa, Iran, Turkey, and the United States, warrants increased sampling of *Rathayibacter* species to determine the distribution of tunicaminyluracil-producing strains, the triggers of toxin production, and the role of tunicaminyluracil in *Rathayibacter* ecology.

## Data Availability Statement

All unpublished genome data have been deposited in GenBank SRA as BioProject PRJNA573074 (CA1 SAMN12797986, CA2 SAMN12797987, CA3 SAMN12797988, CA21 SAMN12797990, CA34 SAMN12797991, CA44 SAMN12797992, CA45 SAMN12797993, CA46 SAMN12797994, CA80 SAMN12797997, CA49 SAMN12797996, CA-47 SAMN12797995, FH164 SAMN12797998, FH176 SAMN12797999, FH 236 SAMN12798000).

## Author Contributions

MT designed the study, analyzed the sequences, and wrote the manuscript draft. AS assisted with sequence analysis. ED performed ANI analysis. TM and BS provided strains and sequences. JC and ER acquired funding and supervised the study. All authors read and approved the final manuscript.

## Disclaimers

Mention of trade names or commercial products in this publication is solely for the purpose of providing specific information and does not imply recommendation or endorsement by the U.S. Department of Agriculture. USDA is an equal opportunity provider and employer.

## Conflict of Interest

The authors declare that the research was conducted in the absence of any commercial or financial relationships that could be construed as a potential conflict of interest.

## Footnotes

1 http://tree.bio.ed.ac.uk/software/figtree/
