# Supplementary material for: The Identification and Conservation of Tunicaminyluracil-Related Biosynthetic Gene Clusters in Several Rathayibacter Species Collected From Australia, Africa, Eurasia, and North America
Source: Front Microbiol. 2020 Jan 10;10:2914. doi: 10.3389/fmicb.2019.02914 (PMC6965331; doi:10.3389/fmicb.2019.02914)
Supplement: Supplementary file 1 [file Table_1.DOCX]

**Supplemental Material**

**TABLE S1**. Overview of *Rathayibacter* strains analyzed in this study.

| Strain | Alternative ID | Species | Sequencing technology (ref)^a^ | Location and year collected | Province/Region | Host | Strain  reference^b^ |
| --- | --- | --- | --- | --- | --- | --- | --- |
| WSM194 | WAC3373 | *R. toxicus* | PacBio (Arif et al., 2016) | Australia, 1978 | Western Australia | *Phalaris paradoxa* | (Arif et al., 2016) |
| FH79/FH137 | 70072 | *R. toxicus* | PacBio/454 (Sechler et al., 2017)  Illumina (Davis et al., 2018) | Australia, 1983 | South Australia | *Lolium rigidum* | (Agarkova et al., 2006) |
| FH81 | 70073 | *R. toxicus* | Illumina (Davis et al., 2018) | Australia, 1973 | Katanning, Western Australia | *Lolium rigidum* | (Agarkova et al., 2006) |
| FH82 | 70074 | *R. toxicus* | Illumina (Davis et al., 2018) | Australia, 1973 | Katanning, Western Australia | *Lolium rigidum* | (Agarkova et al., 2006) |
| FH84 | 70076 | *R. toxicus* | ns | Australia, 1975 | Burra, South Australia | *Lolium rigidum* | (Agarkova et al., 2006) |
| FH85 | 70077 | *R. toxicus* | Illumina (Davis et al., 2018) | Australia, 1975 | Mintaro, South Australia | *Lolium rigidum* | (Agarkova et al., 2006) |
| FH86 | 70078 | *R. toxicus* | Illumina (Davis et al., 2018) | Australia, 1974 | Katanning, Western Australia | *Lolium rigidum* | (Agarkova et al., 2006) |
| FH87 | 70079 | *R. toxicus* | ns | Australia, 1974 | Katanning, Western Australia | *Lolium rigidum* | (Agarkova et al., 2006) |
| FH89 | 70081 | *R. toxicus* | Illumina (Davis et al., 2018) | Australia, 1980 | Gnowangerup, Western Australia | *Lolium rigidum* | (Agarkova et al., 2006) |
| FH128 | 70120 | *R. toxicus* | Illumina (Davis et al., 2018) | Australia, 2001 | Western Australia | *Lolium rigidum* | (Agarkova et al., 2006) |
| FH138 | 70130 | *R. toxicus* | Illumina (Davis et al., 2018) | Australia, 1978 | Western Australia | *Lolium rigidum* | (Agarkova et al., 2006) |
| FH139 | 70131 | *R. toxicus* | Illumina (Davis et al., 2018) | Australia, 1981 | Western Australia | *Phalaris* sp. | (Agarkova et al., 2006) |
| FH140 | 70132 | *R. toxicus* | Illumina (Davis et al., 2018) | Australia, 1983 | Western Australia | *Lolium rigidum* | (Agarkova et al., 2006) |
| FH141 | 70133 | *R. toxicus* | Illumina (Davis et al., 2018) | Australia, 1983 | South Australia | *Lolium rigidum* | (Agarkova et al., 2006) |
| FH142 | 70134 | *R. toxicus* | Illumina (Davis et al., 2018) | Australia, 1981 | Western Australia | *Austrodanthonia caespitosa* | (Agarkova et al., 2006) |
| FH144 | 70136 | *R. toxicus* | ns | Australia, 1981 | Western Australia | *Lolium rigidum* | (Agarkova et al., 2006) |
| FH145 | 70137 | *R. toxicus* | Illumina (Davis et al., 2018) | Australia, 1980 | Western Australia | *Avena sativa* | (Agarkova et al., 2006) |
| FH146 | 70138 | *R. toxicus* | Illumina (Davis et al., 2018) | Australia, 1991 | Western Australia | *Austrodanthonia caespitosa* | (Agarkova et al., 2006) |
| FH147 | 70139 | *R. toxicus* | Illumina (Davis et al., 2018) | Australia, 1984 | South Australia | *Lolium rigidum* | (Agarkova et al., 2006) |
| FH183 | 70175 | *R. toxicus* | Illumina (Davis et al., 2018) | Australia, 1984 | South Australia | *Lolium rigidum* | (Agarkova et al., 2006) |
| FH188 | 70180 | *R. toxicus* | ns | Australia, 1991 | Manoora, South Australia | - | This study |
| FH228 | 70219 | *R. toxicus* | ns | Australia, 1991 | New South Wales | *Agrostis avenacea* | This study |
| FH229 | 70220 | *R. toxicus* | ns | Australia, 1991 | New South Wales | *Agrostis avenacea* | This study |
| FH230 | 70221 | *R. toxicus* | ns | Australia, 1991 | New South Wales | *Agrostis avenacea* | This study |
| FH231 | 70222 | *R. toxicus* | ns | Australia, 1991 | New South Wales | *Agrostis avenacea* | This study |
| FH232/FH100 | 70223 | *R. toxicus* | PacBio (Sechler et al., 2017)  Illumina (Davis et al., 2018) | Australia, 1991 | New South Wales | *Polypogon monspeliensis* | (Agarkova et al., 2006) |
| FH233 | 70224 | *R. toxicus* | ns | Australia, 1991 | New South Wales | *Agrostis avenacea* | This study |
| FH234 | 70225 | *R. toxicus* | ns | Australia, 1991 | New South Wales | *Agrostis avenacea* | This study |
| FH235 | 70226 | *R. toxicus* | ns | Australia, 1991 | New South Wales | *Polypogon monspeliensis* | This study |
| SA03-04 | - | *R. toxicus* | Provided by  Jim Stack | Australia, 2014 | Corny Point, South Australia | *Lolium rigidum* | (Arif et al., 2016) |
| SA03-14 | - | *R. toxicus* | Provided by  Jim Stack | Australia, 2014 | Corny Point, South Australia | *Lolium rigidum* | (Arif et al., 2016) |
| SA03-19 | - | *R. toxicus* | Provided by  Jim Stack | Australia, 2014 | Corny Point, South Australia | *Lolium rigidum* | (Arif et al., 2016) |
| SA08-08 | - | *R. toxicus* | Provided by  Jim Stack | Australia, 2014 | Lake Sunday, South Australia | *Lolium rigidum* | (Arif et al., 2016) |
| SA08-09 | - | *R. toxicus* | Provided by  Jim Stack | Australia, 2014 | Lake Sunday, South Australia | *Lolium rigidum* | (Arif et al., 2016) |
| SA19-02 | - | *R. toxicus* | Provided by  Jim Stack | Australia, 2013 | Yorketown, South Australia | *Lolium rigidum* | (Arif et al., 2016) |
| SA19-06 | - | *R. toxicus* | Provided by  Jim Stack | Australia, 2013 | Yorketown, South Australia | *Lolium rigidum* | (Arif et al., 2016) |
| SA19-07 | - | *R. toxicus* | Provided by  Jim Stack | Australia, 2013 | Yorketown, South Australia | *Lolium rigidum* | (Arif et al., 2016) |
| FH6 | 70005 | *R. iranicus* | PacBio/454 (This study) | Iran, 1966 | - | *Triticum aestivum* | This study |
| FH77 | 70070 | *R. iranicus* | ns | Iran, 1966 | - | *Triticum aestivum* | This study |
| FH154 | 70146 | *R. iranicus* | Illumina (Davis et al., 2018) | Turkey, 2003 | Konya | *Triticum aestivum* | (Postnikova et al., 2009) |
| FH155 | 70147 | *R. iranicus* | ns | Turkey, 2003 | Ankara | *Triticum aestivum* | (Postnikova et al., 2009) |
| FH157 | 70149 | *R. iranicus* | Illumina (Davis et al., 2018) | Turkey, 2003 | Ankara | *Triticum aestivum* | (Postnikova et al., 2009) |
| FH160 | 70152 | *R. iranicus* | ns | Turkey, 2003 | Eskisehir | *Triticum aestivum* | (Postnikova et al., 2009) |
| FH162 | 70154 | *R. iranicus* | ns | Turkey, 2003 | Hisarlikaya | *Triticum aestivum* | (Postnikova et al., 2009) |
| FH163 | 70155 | *R. iranicus* | ns | Turkey, 2003 | Eskisehir | *Triticum aestivum* | (Postnikova et al., 2009) |
| FH164 | 70156 | *R. iranicus* | Illumina  (This study) | Turkey, 2003 | Eskisehir | *Triticum aestivum* | (Postnikova et al., 2009) |
| FH165 | 70157 | *R. iranicus* | ns | Turkey, 2003 | Eskisehir | *Triticum aestivum* | (Postnikova et al., 2009) |
| FH166 | 70158 | *R. iranicus* | ns | Turkey, 2003 | Ankara | *Triticum aestivum* | (Postnikova et al., 2009) |
| FH167 | 70159 | *R. iranicus* | ns | Turkey, 2003 | Eskisehir | *Triticum aestivum* | (Postnikova et al., 2009) |
| FH168 | 70160 | *R. iranicus* | ns | Turkey, 2003 | Eskisehir | *Triticum aestivum* | (Postnikova et al., 2009) |
| FH169 | 70161 | *R. iranicus* | ns | Turkey, 2003 | Ankara | *Triticum aestivum* | (Postnikova et al., 2009) |
| FH170 | 70162 | *R. iranicus* | ns | Turkey, 2003 | Corum | *Triticum aestivum* | (Postnikova et al., 2009) |
| FH173 | 70165 | *R. iranicus* | ns | Turkey, 2003 | Ankara | *Triticum aestivum* | (Postnikova et al., 2009) |
| FH174 | 70166 | *R. iranicus* | ns | Turkey, 2003 | Yaprakbayiri | *Triticum aestivum* | (Postnikova et al., 2009) |
| FH175 | 70167 | *R. iranicus* | ns | Turkey, 2003 | Eskisehir | *Triticum aestivum* | (Postnikova et al., 2009) |
| FH176 | 70168 | *R. iranicus* | Illumina  (This study) | Turkey, 2003 | Ballikuyumcu | *Triticum aestivum* | (Postnikova et al., 2009) |
| FH177 | 70169 | *R. iranicus* | 454  (This study) | Turkey, 2003 | Karaahmet | *Triticum aestivum* | (Postnikova et al., 2009) |
| FH178 | 70170 | *R. iranicus* | ns | Turkey, 2003 | Sarioba | *Triticum aestivum* | (Postnikova et al., 2009) |
| FH179 | 70171 | *R. iranicus* | ns | Turkey, 2003 | Kiranharmani | *Triticum aestivum* | (Postnikova et al., 2009) |
| FH180 | 70172 | *R. iranicus* | ns | Turkey, 2003 | Ankara | *Triticum aestivum* | (Postnikova et al., 2009) |
| CA-1 | - | *R. agropyri* | Illumina  (This study) | USA, 1945 | Montpelier, ID | *Pascopyrum smithii* | (Murray, 1986) |
| CA-2 | - | *R. agropyri* | Illumina  (This study) | USA, 1950 | Muddy Pass, CO | *Elymus trachycaulus* | (Murray, 1986) |
| CA-3 | - | *R. agropyri* | Illumina  (This study) | USA, 1955 | Gilmore, ID | *Elymus lanceolatus* | (Murray, 1986) |
| CA-4 | - | *R. agropyri* | 454  (Davis et al., 2018) | USA, 1986 | Cardwell, MT | *Pascopyrum smithii* | (Schroeder et al., 2018) |
| CA-21 | - | *R. agropyri* | Illumina  (This study) | USA, 2013 | New Meadows, ID | *Pascopyrum smithii* | This study |
| CA-34 | - | *R. agropyri* | PacBio  (This study) | USA, 2013 | White Bird, ID | *Pseudoroegneria spicata* subsp. s*picata* | This study |
| CA-44 | - | *R. agropyri* | Illumina  (This study) | USA, 1950 | Wenatchee, WA | *Elymus bakeri* | This study |
| CA-45 | - | *R. agropyri* | Illumina  (This study) | USA, 1950 | Burns, OR | *Elymus trachycaulus* | This study |
| CA-46 | - | *R. agropyri* | Illumina  (This study) | USA, 1950 | Austin Summit, NV | *Hordeum nodosum* | This study |
| CA-47 | - | *R. agropyri* | Illumina  (This study) | USA, 1950 | Mesa Verde National Park, CO | *Elymus elymoides* | This study |
| CA-49 | - | *R. agropyri* | Illumina  (This study) | USA, 1950 | Colorado | *Poa secunda* | This study |
| CA-80 | - | *R. agropyri* | Illumina  (This study) | USA, 2014 | Grand Coulee, WA | *Leymus cinereus* | This study |
| FH236 | 70227 | *R. woodii* | 454  (This study) | South Africa, 2003 | Western Cape | *Ehrharta villosa* | (Riley et al., 2004) |
| FH237 | 70228 | *R. woodii* | ns | South Africa, 2003 | Western Cape | *Ehrharta villosa* | (Riley et al., 2004) |
| FH238 | 70229 | *R. woodii* | ns | South Africa, 2003 | Western Cape | *Ehrharta villosa* | (Riley et al., 2004) |
| FH239 | 70230 | *R. woodii* | ns | South Africa, 2003 | Western Cape | *Ehrharta villosa* | (Riley et al., 2004) |
| FH240 | 70231 | *R. woodii* | ns | South Africa, 2003 | Western Cape | *Ehrharta villosa* | (Riley et al., 2004) |

^a^The reference for the sequenced *Rathayibacter* strain.

^b^The reference for the identified *Rathayibacter* strain.

Symbols: (ns) = not sequenced, (-) = not known.

**TABLE S3.** Tunicamycin-minimum inhibitory concentration (MIC) values for *Rathayibacter* species.

| *Rathayibacter* species | Strain | MIC-tunicamycin (µg/mL) |
| --- | --- | --- |
| *R. rathayi* | FH95 (70087) | < 0.0625 |
| *R. rathayi* | FH131 (70123) | < 0.0625 |
| *R. rathayi* | FH136 (70128) | < 0.0625 |
| *R. rathayi* | FH129 (70121) | < 0.0625 |
| *R. tritici* | FH212 (70204) | < 0.0625 |
| *R. tritici* | FH5 (70004) | < 0.0625 |
| *R. tritici* | FH96 (70088) | < 0.0625 |
| *R. agropyri* | CA-1 | 8.0 |
| *R. agropyri* | CA-2 | 8.0 |
| *R. agropyri* | CA-3 | < 1.0 |
| *R. agropyri* | CA-4 | 8.0 |
| *R. agropyri* | CA-21 | 8.0 |
| *R. agropyri* | CA-34 | 8.0 |
| *R. agropyri* | CA-44 | 8.0 |
| *R. agropyri* | CA-45 | 8.0 |
| *R. agropyri* | CA-46 | 2.0 |
| *R. agropyri* | CA-47 | 2.0 |
| *R. agropyri* | CA-49 | < 1.0 |
| *R. agropyri* | CA-80 | 2.0 |
| *R. iranicus* | FH6 (70005) | 8.0 |
| *R. iranicus* | FH164 (70156) | 8.0 |
| *R. iranicus* | FH176 (70168) | 8.0 |
| *R. iranicus* | FH177 (70169) | 8.0 |
| *R. toxicus* | FH232 (70223) | 8.0 |
| *R. toxicus* | FH79 (70072) | 8.0 |
| *R. toxicus* | FH145 (70137) | 8.0 |
| *R. woodii* | FH236 (70227) | 8.0 |
| *R. woodii* | FH238 (70229) | 8.0 |

**TABLE S4.** Primers used in study

| Genes (primer) | | Sequence (5’-3’) | Tm (˚C) | Amplicon size (bp) | Reference |
| --- | --- | --- | --- | --- | --- |
| *Rathayibacter toxicus* | |  |  |  | This study |
|  | *tunC* (RtTunC-2) | gcttcagcgccggttctactg | 68 | 1,148 | This study |
|  | *tunC* (RtTunC-1) | gtgaactcgaacgcgggtgagc | 68 | 1,148 | This study |
|  | *tunA* (RtTunA-2) | ctgctgcgcgcgtgcaag | 68 | 1,165 | This study |
|  | *tunA* (RtTunB-1) | ctcgaacaggagcggcgcatg | 68 | 1,165 | This study |
|  | *tunF* (RtTunJ-1) | ggagcgattgcgatcacggtgac | 68 | 1,164 | This study |
|  | *tunF* (TGCtoxicus-R) | gcgctgcacggactccgtctg | 68 | 1,164 | This study |
| *Rathayibacter iranicus* | |  |  |  |  |
|  | *tunC/tunA* (RiTunC-1) | gtgccgcgacacgactgc | 62 | 1,166 | This study |
|  | *tunC/tunA* (RiTunA-1) | cggctcgcgtcaccgtatacttc | 62 | 1,166 | This study |
|  | *tunF/tunQ* (TGCiran-J) | cggcggtcgggtgtgtcg | 66 | 1,719 | This study |
|  | *tunF/tunQ* (TGCiran-T) | cgaggttagtgagccggaagac | 66 | 1,719 | This study |
| *Rathayibacter woodii* | |  |  |  |  |
|  | *tunA-L* (TGCwood-F2) | gtggcgagagcccacagcgc | 75 | 16,670 | This study |
|  | *tunA-L* (TGCwood-R3) | ctgcgacggccacatctagggattc | 75 | 16,670 | This study |

**REFERENCES**

Agarkova, I. V., Vidaver, A. K., Postnikova, E. N., Riley, I. T., and Schaad, N. W. (2006). Genetic Characterization and Diversity of *Rathayibacter toxicus*. *Phytopathology* 96, 1270–1277. doi:10.1094/PHYTO-96-1270.

Arif, M., Busot, G. Y., Mann, R., Rodoni, B., Liu, S., and Stack, J. P. (2016). Emergence of a new population of *Rathayibacter toxicus*: an ecologically complex, geographically isolated bacterium. *PLoS One* 11, e0156182. doi:10.1371/journal.pone.0156182.

Davis, E. W., Tabima, J. F., Weisberg, A. J., Lopes, L. D., Wiseman, M. S., Wiseman, M. S., et al. (2018). Evolution of the US Biological Select Agent, *Rathayibacter toxicus*. *MBio* 9:e01280-1, 1–16.

Murray, T. D. (1986). Isolation of *Corynebacterium agropyri* from 30- to 40-year-old herbarium specimens of *Agropyron* species. *Plant Dis.* 70, 378–380. doi:10.1094/pd-70-378.

Postnikova, E., Agarkova, I., Altundag, S., Eskandari, F., Sechler, A., Karahan, A., et al. (2009). *Rathayibacter iranicus* isolated from symptomless wheat seeds in Turkey. *Plant Pathol.* 58, 796. doi:10.1111/j.1365-3059.2009.02076.x.

Riley, I. T., Swart, A., Postnikova, E., Agarkova, I., Vidaver, A. K., and Schaad, N. W. (2004). New association of a toxigenic *Rathayibacter* sp. and *Anguina woodi* in *Ehrhata villosa* var. *villosa* in South Africa. *Phytopathology* 94, S88.

Schroeder, B. K., Schneider, W. L., Luster, D. G., Sechler, A., and Murray, T. D. (2018). *Rathayibacter agropyri* (non O’Gara 1916) comb. nov., nom. rev., isolated from western wheatgrass (*Pascopyrum smithii*). *Int. J. Syst. Evol. Microbiol.* 68, 1519–1525. doi:10.1099/ijsem.0.002708.

Sechler, A. J., Tancos, M. A., Schneider, D. J., King, J. G., Fennessey, C. M., Schroeder, B. K., et al. (2017). Whole genome sequence of two *Rathayibacter toxicus* strains reveals a tunicamycin biosynthetic cluster similar to *Streptomyces chartreusis*. *PLoS One* 12, 1–17. doi:10.1371/journal.pone.0183005.
